# Evaluation of the Direct Effect of Bilateral Deep Brain Stimulation of the Subthalamic Nucleus on Levodopa-Induced On-Dyskinesia in Parkinson's Disease

**DOI:** 10.3389/fneur.2021.595741

**Published:** 2021-04-12

**Authors:** Jiping Li, Shanshan Mei, Xiaofei Jia, Yuqing Zhang

**Affiliations:** ^1^Beijing Institute of Functional Neurosurgery, Xuanwu Hospital, Capital Medical University, Beijing, China; ^2^Department of Neurology, Xuanwu Hospital, Capital Medical University, Beijing, China

**Keywords:** deep brain stimulation, dyskinesia, Parkinson's disease, subthalamic nucleus, motor complications

## Abstract

**Objective:** This study aimed to evaluate the direct anti-dyskinesia effect of deep brain stimulation (DBS) of subthalamic nucleus (STN) on levodopa-induced on-dyskinesia in Parkinson's disease (PD) patients during the early period after surgery without reducing the levodopa dosage.

**Methods:** We retrospectively reviewed PD patients who underwent STN-DBS from January 2017 to October 2019 and enrolled patients with levodopa-induced on-dyskinesia before surgery and without a history of thalamotomy or pallidotomy. The Unified Dyskinesia Rating Scale (UDysRS) parts I+III+IV and the Unified Parkinson's Disease Rating Scale part III (UPDRS-III) were monitored prior to surgery, and at the 3-month follow-up, the location of active contacts was calculated by postoperative CT–MRI image fusion to identify stimulation sites with good anti-dyskinesia effect.

**Results:** There were 41 patients enrolled. The postoperative levodopa equivalent daily dose (LEDD) (823.1 ± 201.5 mg/day) was not significantly changed from baseline (844.6 ± 266.1 mg/day, *P* = 0.348), while the UDysRS on-dyskinesia subscores significantly decreased from 24 (10–58) to 0 (0–18) [median (range)] after STN stimulation (*P* < 0.0001). The levodopa-induced on-dyskinesia recurred in stimulation-off/medication-on state in all the 41 patients and disappeared in 39 patients when DBS stimulation was switched on at 3 months of follow-up. The active contacts which correspond to good effect for dyskinesia were located above the STN, and the mean coordinate was 13.05 ± 1.24 mm lateral, −0.13 ± 1.16 mm posterior, and 0.72 ± 0.78 mm superior to the midcommissural point.

**Conclusions:** High-frequency electrical stimulation of the area above the STN can directly suppress levodopa-induced on-dyskinesia.

## Introduction

Dyskinesia is one of the most troublesome symptoms of advanced Parkinson's disease (PD), often induced by long-term dopaminergic treatment (levodopa-induced dyskinesia, LID). Following the definition in the Unified Dyskinesia Rating Scale (UDysRS) (1), LID is divided into two types: (1) on-dyskinesia and (2) off-dystonia. On-dyskinesia, which refers to the choreic and dystonic movements that occur when medicine is working (1), is present in 70–80% of PD patients who experience dyskinesia (2).

Deep brain stimulation (DBS) of subthalamic nucleus (STN) could reduce the required levodopa dosage for symptom control (3, 4), and the majority of researchers opine that the anti-dyskinesia effect of STN stimulation is mainly due to the significant postoperative reduction of levodopa medication (5–8), which is an indirect inhibition. However, Kim et al. found that LID was reduced following STN-DBS in PD regardless of whether the levodopa dosage was reduced (9), and by developing a multiple regression model to predict postoperative dyskinesia scores, Mossner et al. found that STN-DBS improved dyskinesia beyond levodopa reduction (10). In addition, some data suggested that STN-DBS may also have direct anti-dyskinesia effect (9, 11–15). In our center, we switch on the stimulation within 3 days after DBS implantation without levodopa dosage reduction till the first follow-up at 3 months postoperatively, which provides an opportunity to evaluate the direct anti-dyskinesia effect of STN-DBS.

The DBS strategies could be different for on-dyskinesia and off-dystonia: stimulating the sensorimotor region could significantly improve cardinal parkinsonian symptoms (tremor, rigidity, and bradykinesia) (16) and also significantly improve off-dystonia (6, 17), while stimulating STN itself could not suppress on-dyskinesia (11) and even induce dyskinesia (18–20); therefore, this study only focuses on levodopa-induced on-dyskinesia. We retrospectively reviewed the changes of on-dyskinesia without medication reduction during the first 3 months postoperatively to evaluate the direct anti-dyskinesia effect of STN-DBS on levodopa-induced on-dyskinesia and tried to identify stimulation sites with good anti-dyskinesia effect.

## Methods

### Subjects

We retrospectively reviewed the clinical records of 146 PD patients who underwent STN-DBS by the same two neurosurgeons (Zhang and Li) at the Xuanwu Hospital of Capital Medical University from January 2017 to October 2019. Patients who suffered from preoperative levodopa-induced on-dyskinesia and with a score of Unified Parkinson's Disease Rating Scale (UPDRS) (part IV, item 32) ≥1 were included, and patients who had a history of thalamotomy or pallidotomy, which may suppress LID, were excluded. Eventually, 41 patients were included in this study. Of the 41 patients, 23 were female and 18 were male. Their mean age was 62.7 ± 8.2 years. The mean duration of disease before the surgery was 10.4 ± 3.7 years. Forty patients presented with peak-dose dyskinesia and 1 patient (P2) with square-wave dyskinesia. Thirty-three patients had bilateral on-dyskinesia and 8 patients had unilateral dyskinesia at baseline (Supplementary Table 1). All these patients met the MDS diagnostic criteria of PD and had bilateral STN-DBS implantation. The study was approved by the Ethics Committee of Xuanwu Hospital of Capital Medical University.

### DBS Surgical Procedure and Coordinates of DBS Electrode

DBS electrode implantation was performed under local anesthesia. The CRW stereotactic frame (Radionics, Webster, New York, USA) was applied under local anesthesia, then CT scanning was performed. The CT images were fused immediately with the preoperative magnetic resonance imaging (MRI; Siemens 3.0 Tesla, Sonata, Germany) images through the StealthStation Surgical Navigation System (Medtronic, Minneapolis, Minnesota, USA), and the coordinates of the target and the entrance trajectory were defined on stereotactic MRI images by directly visualizing the STN. Intraoperative microelectrode single needle recording (MER) using the Microdrive system (Alpha Omega Engineering, Nazareth, Israel) was performed, starting from 10 mm above the target. After the precise localization of the target point, DBS electrodes (Model 3389, Medtronic, Minneapolis, MN, USA) with four contacts were placed in such a way that the metal tip of the DBS electrode was located 2–3 mm above the ventral STN border, and the contacts were positioned and labeled as follows: contacts 0 and 1, inside the STN; contact 2, dorsal margin of the STN; and contact 3, above the STN. Then, the DBS electrodes were tunneled and connected to a rechargeable implantable pulse generator (Activa® RC, Medtronic, Minneapolis, MN, USA) implanted in the subclavian region under general anesthesia. Postoperative CT images were fused with the preoperative MRI images to confirm the final position of the electrode metal tip and the trajectory of the DBS electrode and to calculate the coordinates of each contact, and the distance from the metal tip to the center of each contact (distal to proximal: contact 0, contact 1, contact 2, and contact 3) was 0.75, 2.75, 4.75, and 6.75 mm, respectively.

### DBS Programming

DBS programming was initiated within 3 days after surgery with an initial setting of 60–90 μs/130–160 Hz/1.0–1.5 V. Patients underwent adjustment of stimulation settings until optimal control of the symptoms was established during hospitalization. The adjustment strategy of DBS programming was as follows: firstly, we used unipolar stimulation and chose the contact which positioned at the dorsal margin of the STN as the active contact for patients with LID and the contact inside the STN for patients without LID; if the patient did not achieve good control of symptoms, then it was changed to dual-contact monopolar stimulation (a contact within the STN + a contact above the STN); finally, interleaving stimulation was utilized, when necessary.

Patients had the first postoperative clinical assessments and adjustment of stimulation settings and medication in the 3-month follow-up. On the 1st day of follow-up, the stimulation parameters were carefully screened following all contacts in medication-off (Med-off) state after at least 12 h without taking any anti-parkinsonian medication in the morning; the contacts and stimulation parameters were optimized to obtain maximum clinical benefit and minimal side effects. After switching off DBS for 30 min, patients took the usual first morning dose of levodopa; if on-dyskinesia occurred, then we switched on the DBS to test the anti-dyskinesia effect of active contacts.

### Clinical Assessment and Statistical Analysis

The outcome assessments consisted of the on-dyskinesia subscores of the UDysRS (parts I+III+IV) and UPDRS-III before surgery and 3 months after surgery. Baseline assessments of UPDRS-III were completed in Med-off state after at least 12 h without taking any anti-parkinsonian medication, and UPDRS-III of the Med-on state was the maximum improvement following a dose of levodopa equal to 150% of the patient's usual first morning dose. At the 3-month follow-up, all scores were assessed in DBS stimulation-on (Stim-on) condition on the 2nd day following the same dose of levodopa as baseline. The clinical improvement was computed as ([(Prescores – Postscores)/Prescores] ^*^ 100%). Student's *t*-test or the Wilcoxon signed-rank test was used to determine whether there was a significant difference between the clinical scale scores at baseline and at 3 months follow-up. Statistical analysis was performed with SPSS (version 20.0; SPSS Inc, Chicago, IL). *P* < 0.05 were considered statistically significant.

## Results

### Clinical Outcome

**Figure 1:**
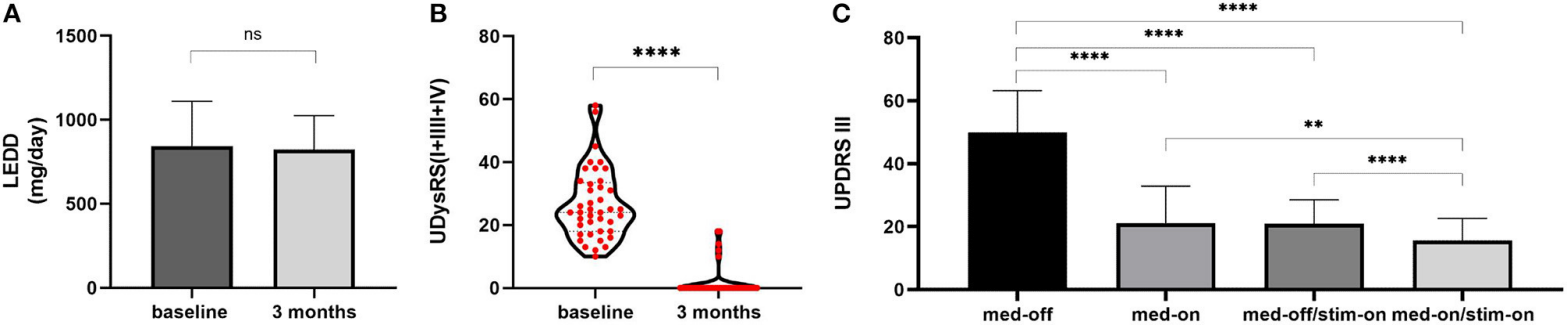
LEDD changes and outcome of UDysRS and UPDRS-III. **(A)** LEDD: at baseline 844.6 ± 266.1 mg/day, at 3 months of follow-up 823.1 ± 201.5 mg/day. **(B)** Violin with plots showing UDysRS (I+III+IV) scores: 24 (10–58) at baseline to 0 (0–18) at 3 months of follow-up. **(C)** UPDRS-III: Med-off 49.95 ± 13.30, Med-on 21.03 ± 11.87 at baseline; Med-off/Stim-on 20.88 ± 7.68, Med-on/Stim-on 15.55 ± 7.11 at 3 months of follow-up. The values are presented as mean ± standard deviation or median (range). ****, *P* < 0.0001; **, *P* < 0.01; ns, non-significant; LEDD,levodopa equivalent daily dose; UDysRS, Unified Dyskinesia Rating Scale; UPDRS-III, Unified Parkinson's Disease Rating Scale part III.

The postoperative levodopa equivalent daily dose (LEDD) (823.1 ± 201.5 mg/day) was not significantly changed from the baseline (844.6 ± 266.1 mg/day, *P* = 0.348) (Figure 1A). There were 39 patients without levodopa dosage reductions, and 2 patients (P38, P40) with the addition of amantadine and a reduction of LEDD for persistent dyskinesia after surgery.

However, the UDysRS on-dyskinesia subscores significantly reduced after STN-DBS stimulation [from baseline 24 (10–58) to 0 (0–18), median (range), *P* < 0.0001; Figure 1B); 36/41 (87.8%) patients scored 0 on UPDRS-IV item 32, and only 5 patients (P27, P28, P30, P38, P40) continued to experience persistent dyskinesia, and their dyskinesia was observed in four experimental conditions with stimulation and medication on and off subdivided into the following (Table 1): 1 patient (P27) presented with stimulation-induced dyskinesia (SID), 2 patients (P28, P30) presented with unilateral levodopa-induced on-dyskinesia, and the remaining 2 patients (P38, P40) experienced abnormal involuntary movements after DBS surgery despite medication withdrawal and cessation of DBS stimulation, which may be induced by a microlesion in the STN due to surgery (surgery-related dyskinesia, SRD) (21). In other words, levodopa-induced on-dyskinesia was completely relieved in 39/41 (95%) patients.

**Table 1:** Body parts involved by dyskinesia.

**Patients**	**On-dyskinesia at baseline**	**Dyskinesia at 3-month follow-up**				**Type of postoperative dyskinesia in Stim-on**
		**Med-off/Stim-off**	**Med-on/Stim-off**	**Med-off/Stim-on**	**Med-on/Stim-on**	
P27	Left upper limb	–	Left upper limb	Left foot	Left foot	SID
P28	Left limbs and right upper limb	–	Left limbs and right upper limb	–	Right upper limb	LID
P30	Upper limbs	–	Upper limbs	–	Left upper limb	LID
P38	Four limbs and trunk	Right foot	Four limbs	Right foot	Right foot	SRD
P40	Neck, four limbs, and trunk	Right foot	Four limbs and trunk	Right foot	Right foot	SRD

*SID, stimulation-induced dyskinesia; LID, levodopa-induced dyskinesia; SRD, surgery-related dyskinesia*.

There were a 57.5 ± 14.5% improvement in UPDRS-III scores in Med-off/Stim-on state relative to the Med-off state at baseline (from 49.95 ± 13.30 to 20.88 ± 7.68, *P* < 0.0001) and a 69.0 ± 12.4% improvement in Med-on/Stim-on relative to the Med-off at baseline (from 49.95 ± 13.30 to 15.55 ± 7.11, *P* < 0.0001) (Figure 1C).

### Coordinates of Electrode and Programming Settings

**Figure 2:**
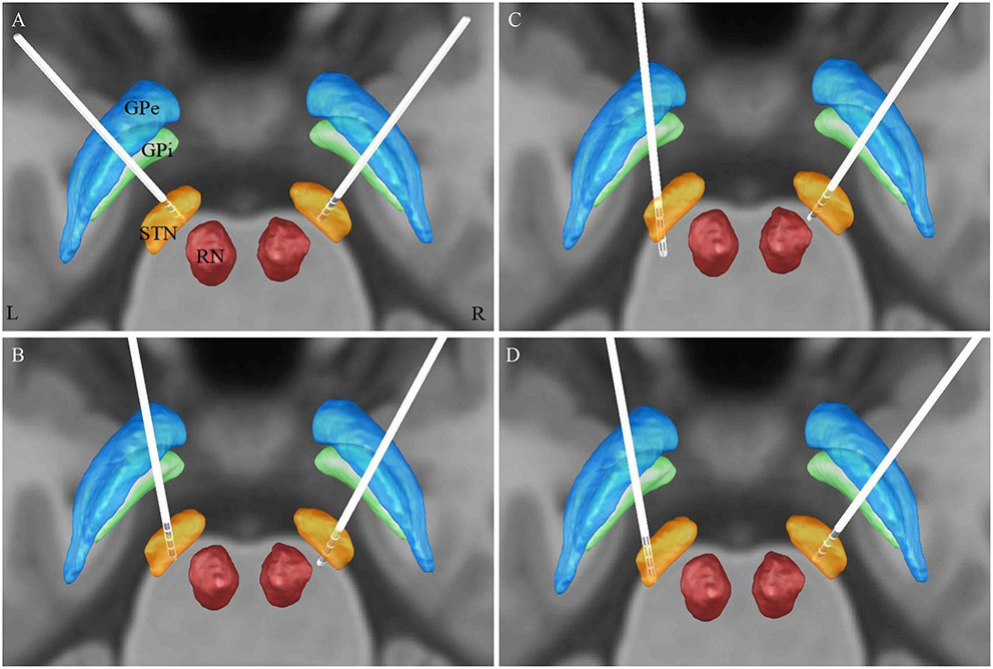
3D illustration for the localization of electrode contacts (Model 3389, Medtronic) by lead-DBS software: **(A)** patient 28, **(B)** patient 30, and **(C)** patient 41 (initial implantation): the left electrode of P28, the right electrode of P30, and the bilateral electrodes of P41 were implanted deeper than planning, and the most dorsal contact (contact 3) was located inside the STN; on the other hand, the right electrode of P28 and the left electrode of P31 were implanted as planning: contacts 0 and 1 were located inside the STN, contact 2 was located at the dorsal margin of the STN, and contact 3 was located above the STN. **(D)** patient 41 (after relocation).

Four electrodes were implanted deeper than planning: the left electrode of P28, the right electrode of P30, and the bilateral electrodes of P41 (Figures 2A–C), and the vertical coordinates (*Z*-axis) of the electrode metal tip were −7.50, −7.71, −8.31, and −7.65 mm, respectively, inferior to the midcommissural point. P41 underwent dorsal relocation of bilateral DBS electrodes on the 6th day postoperatively by withdrawing the left DBS electrode 4 mm and the right electrode 2 mm (Figure 2D). The final coordinates of the electrode metal tip relative to the midcommissural point are described in Table 2.

**Table 2:** Position of the electrodes and DBS settings.

**Localization and DBS settings**		**Left electrode**	**Right electrode**
Coordinates of the electrode metal tip relative to the midcommissural point (mm)	Lateral (*X*-axis)	−11.55 ± 1.22 (−8.38 to −14.05)	11.21 ± 1.21 (8.49~14.48)
	Anteroposterior (*Y*-axis)	−2.37 ± 1.07 (−0.56 to −4.90)	−2.72 ± 1.07 (−0.74 to −4.64)
	Vertical (*Z*-axis)	−4.91 ± 1.15 (−1.86 to −7.50)	−5.16 ± 0.78 (−3.65 to −7.71)
Stimulation parameter	Frequency (Hz)	145.73 ± 15.63 (120~160)	145.73 ± 15.62 (120~160)
	Pulse widths (μs)	85.12 ± 13.25 (60~120)	84.15 ± 13.96 (60~120)
	Amplitudes (V)	2.17 ± 0.38 (1.5~3.0)	2.23 ± 0.40 (1.5~3.0)

*The values are presented as mean ± standard deviation (minimum–maximum)*.

**Figure 3:**
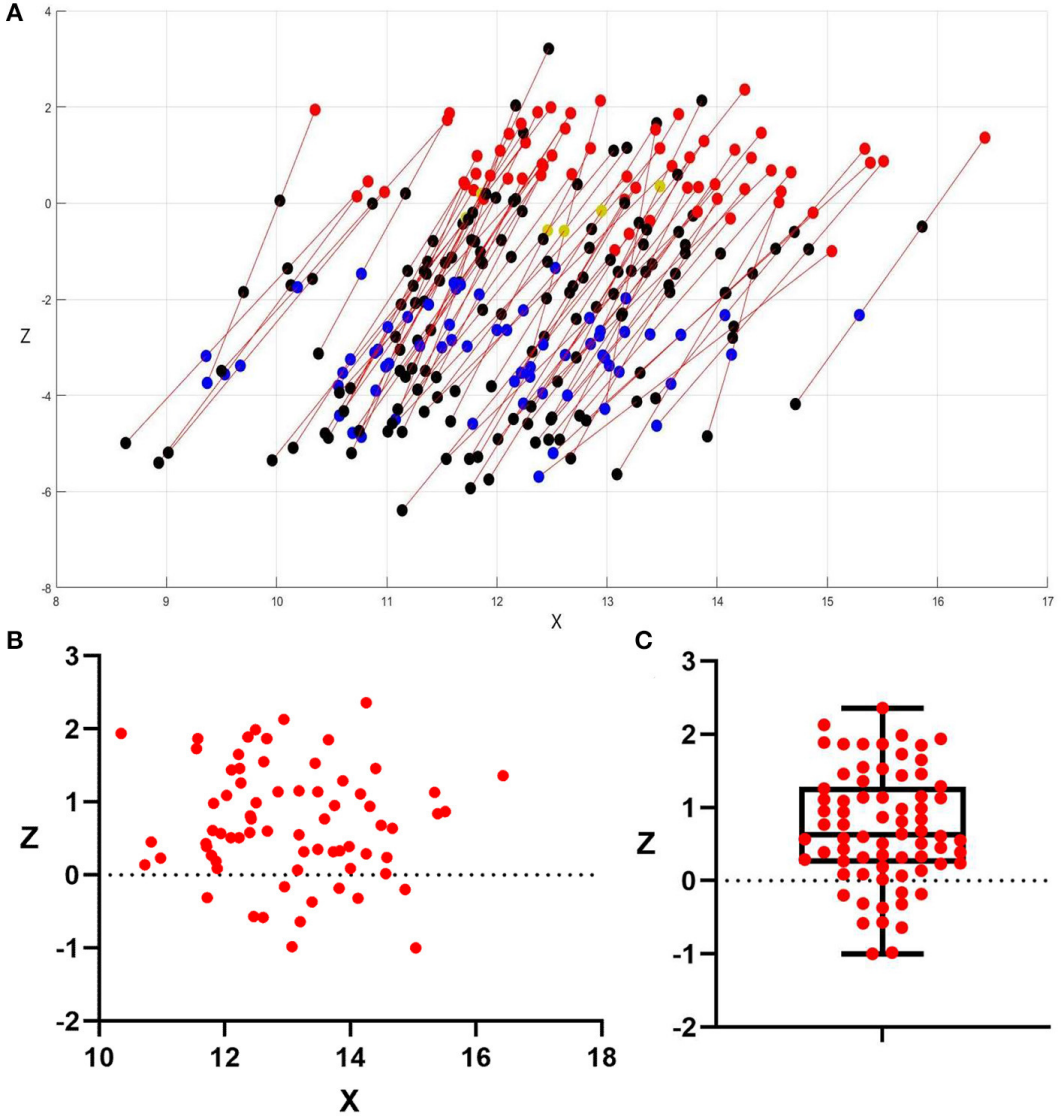
Distribution of electrode and active contacts. **(A)** Distribution of 72 electrodes which got good LID management, and all DBS electrodes were mapped to the right side to allow for direct comparison. The *X*-coordinate is positive toward the lateral. Unipolar stimulation in 6 electrodes (yellow plots are active contacts) and double monopolar stimulation or interleaving stimulation in 66 electrodes (red and blue plots are active contacts); red plots and yellow plots were the contacts corresponding to good anti-dyskinesia effect, while blue plots were the contacts corresponding to good effect for PD motor symptoms but without anti-dyskinesia effect; black plots were inactive contacts. **(B)** Distribution of the 72 active contacts which showed successful anti-dyskinesia effect, 2D diagram in an anterior view; **(C)** *Z*-coordinate of the 72 active contacts which showed successful anti-dyskinesia effect: 0.72 ± 0.78 mm (−1~2.36 mm). The values were presented as mean ± standard deviation (minimum–maximum); 61/72 (85%) were above the anteroposterior commissure plane (*Z* = 0).

DBS programing settings are summarized in Table 2 and Supplementary Table 1. A total of 74 STN-DBS electrodes were programmed for levodopa-induced on-dyskinesia management, and a complete relief of such dyskinesia was found in 72 electrodes (Figure 3A): dual-contact monopolar stimulation or interleaving stimulation (two active contacts: C+1–3– or C+0–3– or C+0–2–) was utilized in 66 electrodes and unipolar stimulation (C+2–) was utilized in 6 electrodes.

At 3 months follow-up, all the patients still presented with choreatic on-dyskinesia in Med-on/Stim-off condition; by testing the effort of these 138 active contacts, we found that the direct suppression of levodopa-induced on-dyskinesia was achieved by the stimulation of dorsal contacts (contact 3 in 65 electrodes and contact 2 in 7 electrodes), which were located above 1 mm inferior to the anteroposterior commissure plane (*Z* = −1); the mean coordinate of these 72 contacts was 13.05 ± 1.24 mm lateral, −0.13 ± 1.16 mm posterior, and 0.72 ± 0.78 mm superior to the midcommissural point, and the majority (85%) were above the anteroposterior commissure plane (Figures 3B,C). The unipolar stimulation (C+3–) was utilized in two electrodes (left electrode of P28 and right electrode of P30) but failed to suppress the contralateral LID; this two contacts were located within STN (Figures 2A,B).

### Adverse Events

There were 55 contacts of 31 electrodes (19 patients) that were found to induce dyskinesia (SID) (Supplementary Table 1), which were located inside the STN. The SID was completely relieved by changing to dorsal contact stimulation or dual-contact monopolar stimulation in 18 patients, while 1 patient experienced persistent SID (P27) at 3 months postoperatively. There were four patients (P37–40) who experienced SRD: in two patients, SRD self-resolved before 3 months follow-up (P37, P39), while it persisted in the remaining two patients (P38, P40). Infection of the incision occurred in one patient (P25).

### Case Description for Special Cases

There was one patient (P41) who continued to experience persistent bilateral levodopa-induced on-dyskinesia after DBS stimulation by using the most dorsal contacts, even reducing the medication from Madopar 125 mg every 4 hours(q4h) to 62.5 mg q4h, and the coordinates of the electrode metal tip relative to the midcommissural point were left (*X, Y, Z* −12.35, −5.74, −8.31 mm) and right (10.85, −2.59, −7.65 mm). Bilateral DBS electrodes were dorsally repositioned under a local anesthetic on the 6th day postoperatively by withdrawing the left DBS electrode 4 mm and the right electrode 2 mm, and there was sustained relief of dyskinesia using the most dorsal contacts without levodopa reduction (Figures 2C,D).

At 3 months follow-up, there were five patients (P27, P28, P30, P38, and P40) who continued to experience persistent dyskinesia: SRD for two patients (P38, P40) and it finally self-resolved between 4 and 6 months postoperatively; SID for one patient (P27) and it was completely relieved after 1 year postoperatively when his tremor became less prominent and a good effect was obtained under the SID threshold; and the remaining two patients had unilateral levodopa-induced on-dyskinesia (P28, P30), which was finally completely relieved after levodopa reduction.

## Discussion

In our study, from the overall level, we found that the LEDD after surgery was not significantly changed from baseline, but UDysRS on-dyskinesia subscores significantly decreased; from the individual level, we found that levodopa-induced on-dyskinesia recurred in Stim-off/Med-on state and disappeared when DBS stimulation was switched on in 39/41 (95%) patients at the 3-month follow-up. All these findings confirm that STN-DBS stimulation can directly suppress levodopa-induced on-dyskinesia.

The key point is which specific region of STN or around STN is responsible for the direct anti-dyskinesia effect. We found that stimulating STN itself could not suppress on-dyskinesia, even induce dyskinesia, which is consistent with previous reports (11, 18–20). The active contacts which correspond to good anti-dyskinesia effect in our study were all located above 1 mm inferior to the anteroposterior commissure plane (*Z* = −1), where the dorsal margin of the STN is estimated by microrecording (4, 22). This finding suggests that stimulation above the STN can result in direct suppression of on-dyskinesia. Several previous studies, by superimposing the location of the electrodes onto the Schaltenbrand–Wahren atlas (13) or using the volume of tissue-activated models (23), had the same findings. The above STN area is a complex area between the dorsal STN border and the ventral thalamus (23), including the zona incerta (24) and Forel's field H (25), where pallidothalamic, pallidosubthalamic, or subthalamopallidal fibers are densely distributed (13). Stimulation of these fibers may cause similar effects to pallidal DBS and, therefore, directly suppress dyskinesia (11–14). In addition, we found the majority (86%) of these active contacts located above the anteroposterior commissure plane (*Z* = 0), and the average vertical coordinate (*Z*-axis) was 0.72 mm superior to the midcommissural point (*Z* = +0.72), which is dorsally compared with the dorsal margin of the STN and consistent with Yoichi's observations (12). It suggests that the dorsal portion of above STN area may have a more definite anti-dyskinesia effect.

The anti-dyskinesia effect of STN-DBS in our study is much better than that reported in previous literature, which is mainly due to our strategy to implant Medtronic 3389 DBS electrode 2–3 mm above compared with the conventional procedure as described previously (26, 27). STN-DBS could not achieve a good anti-dyskinesia effect probably because the DBS electrode was implanted too deep to provide adequate coverage of the above STN. That is what happened to the four electrodes that were implanted deeper than planning, and two of them, which were able to suppress dyskinesia after dorsal relocation, confirmed it also. Thus, the depth of electrode insertion for STN-DBS is the crucial point for dyskinesia suppression. What is more, the hot spot for optimal improvement of motor symptoms of PD was dorsal to the center of the STN, but within STN boundaries (22, 28, 29). Hence, we implanted the DBS electrode in such a way that the metal tip of the electrode was positioned 2–3 mm above the ventral margin of the STN, to ensure that the contacts cover the dorsal two-thirds portion of the STN [the motor region of the STN (30)] and above STN area, and the clinical outcome suggests that this implantation is effective.

Also (and this is important!), we carefully observed the relationship between dyskinesia, medication, and DBS stimulation to subdivide the type of postoperative dyskinesia. After STN-DBS, especially during the early postoperative period, dyskinesia could be complicated. Besides LID, two new types of dyskinesia came out: (1) SID (18–20), which is defined as abnormal involuntary movements that occur when stimulation is on and disappear when stimulation is off; 46.3% (19/41) of patients in our study developed SID, while SID was completely relieved in 95% (18/19) of patients by adjustment of DBS stimulation settings. (2) SRD, which persisted despite levodopa withdrawal (Med-off) and cessation of stimulation (Stim-off) after STN-DBS surgery, may be induced by a microlesion in the STN and self-resolved in several weeks or months (21). Four patients in our study suffered from SRD and it self-resolved between 2 weeks and 6 months. The two types of dyskinesia were considered transient adverse effects of STN-DBS surgery, but they usually predict a good outcome of DBS (18–21). In our study, postoperative dyskinesia all presented choreatic abnormal involuntary movements; since the same expression of dyskinesia may have different etiologies and different treatment strategies, it is important to subdivide postoperative dyskinesia for the evaluation of the effect of STN-DBS on a certain type of dyskinesia and to determine an appropriate treatment strategy.

## Conclusions

High-frequency electrical stimulation of the area above the STN can directly suppress levodopa-induced on-dyskinesia, and the STN-DBS strategy for PD patients with levodopa-induced on-dyskinesia is simultaneously stimulating both the sensorimotor region of the STN and the area above the STN. The depth of electrode insertion for STN-DBS to provide adequate coverage of the above STN is the crucial point for dyskinesia suppression.

## Data Availability Statement

The original contributions presented in the study are included in the article/Supplementary Material, further inquiries can be directed to the corresponding author/s.

## Ethics Statement

The studies involving human participants were reviewed and approved by the ethics committee of Xuanwu Hospital of Capital Medical University. The patients/participants provided their written informed consent to participate in this study.

## Author Contributions

JL was the major contributor in writing the manuscript and contributed to the DBS programming. SM contributed to the diagnosis and clinical assessment of the patients. JL and YZ contributed to DBS surgery. XJ contributed to data acquisition. SM and YZ contributed to the manuscript editing. YZ was the guarantor of integrity of the entire study. All the authors had collectively poured in a lot of efforts into this study, read, and approved the final manuscript.

## Conflict of Interest

The authors declare that the research was conducted in the absence of any commercial or financial relationships that could be construed as a potential conflict of interest.
