# Supplementary material for: Evaluation of the Direct Effect of Bilateral Deep Brain Stimulation of the Subthalamic Nucleus on Levodopa-Induced On-Dyskinesia in Parkinson's Disease
Source: Front Neurol. 2021 Apr 12;12:595741. doi: 10.3389/fneur.2021.595741 (PMC8072270; doi:10.3389/fneur.2021.595741)
Supplement: Supplementary file 1 [file Table_1.docx]

| patients | side of LID  B/R/L | contacts induce SID | Parameters of left electrode | | | | Parameters of right electrode | | | |
| --- | --- | --- | --- | --- | --- | --- | --- | --- | --- | --- |
|  |  |  | Stimulation mode | Pulse Width, (μs) | Frequency (Hz) | Voltage  (V) | Stimulation mode | Pulse Width, (μs) | Frequency (Hz) | Voltage  (V) |
| P1 | B | --- | C+8-11- | 90 | 160 | 2.0 | C+0-3- | 90 | 160 | 2.0 |
| P2 | B | --- | C+9-11- | 60 | 160 | 2.2 | C+1-3- | 60 | 160 | 2.0 |
| P3 | L | --- | C+9- | 90 | 130 | 2.0 | C+1-3- | 60 | 130 | 1.5 |
| P4 | R | --- | C+9-11- | 90 | 130 | 2.3 | C+1- | 60 | 130 | 2.2 |
| P5 | B | --- | C+9-11- | 60 | 160 | 2.0 | C+1-3- | 60 | 160 | 2.0 |
| P6 | B | --- | C+10 | 90 | 160 | 2.8 | C+2- | 90 | 160 | 2.2 |
| P7 | B | --- | C+9-11- | 60 | 130 | 2.5 | C+1-3- | 90 | 130 | 2.3 |
| P8 | B | --- | C+9-11- | 90 | 130 | 3.0 | C+1-3- | 90 | 130 | 2.5 |
| P9 | B | --- | C+10 | 90 | 160 | 2.7 | C+2- | 90 | 160 | 2.7 |
| P10 | B | --- | C+9-11- | 70 | 160 | 2.4 | C+1-3- | 70 | 160 | 2.3 |
| P11 | B | --- | C+8-11- | 90 | 160 | 1.9 | C+1-3- | 80 | 160 | 1.8 |
| P12 | B | --- | C+8-11- | 90 | 160 | 2.0 | C+1-3- | 90 | 160 | 2.2 |
| P13 | B | --- | C+9-11- | 80 | 130 | 2.25 | C+0-3- | 90 | 130 | 2.20 |
| P14 | L | --- | C+9- | 90 | 130 | 2.7 | C+1-3- | 90 | 130 | 2.5 |
| P15 | B | --- | C+8-11- | 90 | 130 | 2.8 | C+0-3- | 90 | 130 | 2.8 |
| P16 | R | --- | C+9-11- | 90 | 160 | 2.0 | C+1- | 90 | 160 | 2.5 |
| P17 | B | --- | C+9-11- | 90 | 160 | 2.0 | C+1-3- | 90 | 160 | 2.0 |
| P18 | B | --- | C+9-11- | 60 | 160 | 2.0 | C+0-3- | 60 | 160 | 1.8 |
| P19 | B | --- | C+9-11- | 60 | 160 | 1.8 | C+0-3- | 60 | 160 | 2.6 |
| P20 | B | --- | C+8-10- | 90 | 160 | 2.0 | C+1-3- | 90 | 160 | 1.5 |
| P21 | B | 0/8,9 | C+8-11- | 90 | 160 | 2.0 | C+1-3- | 90 | 160 | 2.0 |
| P22 | B | 0/8 | C+9-11- | 90 | 130 | 2.3 | C+1-3- | 90 | 130 | 2.0 |
| P23 | B | 0,1/8,9 | C+9-11- | 60 | 160 | 1.8 | C+1-3- | 70 | 160 | 1.8 |
| P24 | B | 8,9 | C+9-11- | 90 | 130 | 2.8 | C+1-3- | 90 | 130 | 2.5 |
| P25 | L | 0,1/8 | C+8-11- | 100 | 160 | 1.5 | C+1-3- | 100 | 160 | 1.8 |
| P26 | B | 8,9 | C+9-11- | 60 | 160 | 2.5 | C+1-3- | 90 | 160 | 2.2 |
| P27 | L | 0,1/8,9 | C+10- | 90 | 160 | 2.7 | C+1-3- | 90 | 160 | 2.5 |
| P28 | B | 8,9,10 | C+11- | 80 | 130 | 2.4 | C+1-3- | 90 | 130 | 2.5 |
| P29 | B | 0,1/8,9 | C+9-11- | 90 | 130 | 2.0 | C+1-3- | 90 | 130 | 2.0 |
| P30 | B | 0,1,2/8 | C+8-11- | 90 | 160 | 1.8 | C+3- | 90 | 160 | 2.2 |
| P31 | B | 0,1/8,9 | C+9-11- | 90 | 130 | 1.7 | C+1-3- | 90 | 130 | 1.6 |
| P32 | B | 0/8,9 | C+8-  C+11- | 90  90 | 120 | 1.5  2.0 | C+0-  C+3- | 90  90 | 120 | 1.5  2.0 |
| P33 | B | 0,1/8,9 | C+9-11- | 90 | 130 | 2.5 | C+1-3- | 90 | 130 | 1.5 |
| P34 | B | 0,1/8,9 | C+9-  C+11- | 90  90 | 125 | 2.0  2.3 | C+1-  C+3- | 90  90 | 125 | 2.3  3.0 |
| P35 | L | 8 | C+9- | 90 | 160 | 2.8 | C+1-3- | 60 | 160 | 2.3 |
| P36 | B | 8 | C+9-11- | 90 | 160 | 2.0 | C+1-3- | 90 | 160 | 1.6 |
| P37 | L | --- | C+8- | 90 | 130 | 1.6 | C+1-3- | 90 | 130 | 2.5 |
| P38 | B | 8,9 | C+9-11- | 90 | 130 | 2.7 | C+1-3- | 120 | 130 | 2.7 |
| P39 | B | 8,9 | C+8-11- | 90 | 130 | 2.6 | C-0-3- | 60 | 130 | 2.5 |
| P40 | B | 0,1/8,9 | C+10- | 100 | 130 | 2.5 | C+2- | 100 | 130 | 2.5 |
| P41 | B | -- | C+9-11- | 120 | 160 | 2.75 | C+1-3- | 90 | 160 | 2.2 |

Table S1. DBS Programming Parameters at 3 months follow-up. B/R/L, bilateral/right/left; SID, stimulation induced dyskinesia.
